# Elevated propionate and its association with neurological dysfunctions in propionic acidemia

**DOI:** 10.3389/fnmol.2025.1499376

**Published:** 2025-03-19

**Authors:** Xiaoxin Chen, Qing Cheng, Guo-Fang Zhang

**Affiliations:** ^1^Surgical Research Lab, Department of Surgery, Cooper University Hospital, Cooper Medical School of Rowan University, Camden, NJ, United States; ^2^Coriell Institute for Medical Research, Camden, NJ, United States; ^3^MD Anderson Cancer Center at Cooper, Camden, NJ, United States; ^4^Biomedical/Biotechnology Research Institute, North Carolina Central University, Durham, NC, United States; ^5^Sarah W. Stedman Nutrition and Metabolism Center, Duke Molecular Physiology Institute, Duke University, Durham, NC, United States; ^6^Division of Endocrinology, Metabolism and Nutrition, Department of Medicine, Duke University Medical Center, Durham, NC, United States

**Keywords:** propionic acidemia, propionate, neurological dysfunction, microbiome, short-chain fatty acids, rare metabolic diseases, metabolism

## Abstract

Propionate, a short-chain fatty acid (SCFA), has recently attracted attention for its various health benefits. However, elevated levels of propionate in certain pathological conditions can have adverse effects. Propionic acidemia (PA) is a rare metabolic disorder caused by mutations in the propionyl-CoA carboxylase (PCC) gene (*PCCA* or *PCCB*), leading to reduced PCC activity and impaired propionyl-CoA metabolism. This metabolic block at the PCC-mediated step results in the accumulation of propionyl-CoA and its metabolites, including propionate, contributing to various complications, such as neurological dysfunction, in patients with PA. This review examines propionate synthesis, its physiological role, its metabolism in healthy individuals and those with PA, and the pathological link between elevated propionate levels and neurological dysfunctions in PA patients. A deeper understanding of propionate metabolism under both normal and pathological conditions will help clarify the full spectrum of its metabolic effects.

## 1 Introduction

Propionate, a short-chain fatty acid (SCFA) produced by the microbiome, is chemically similar to acetate but follows a distinct metabolic pathway. While acetate supports energy metabolism and fatty acid synthesis through the provision of acetyl-CoA, propionate acts as an anaplerotic substrate, replenishing tricarboxylic acid (TCA) cycle intermediates when cataplerosis depletes them (Wolever et al., 1991; Brunengraber and Roe, 2006). The metabolic flux of propionyl-CoA entering the TCA cycle is significantly slower than that of acetyl-CoA (Wang et al., 2024). However, anaplerosis is essential in organs with high cataplerotic activity, such as the liver, kidneys, intestines, pancreas, and brain (Weidemann et al., 1970; Mithieux and Gautier-Stein, 2014; Zhang et al., 2016; He et al., 2021; Marin-Valencia et al., 2024). Moreover, propionate supplementation has been shown to offer benefits beyond its role in anaplerosis, such as reducing lipogenesis, lowering serum cholesterol levels, mitigating depressive-like behavior, and reducing carcinogenesis risk (Hosseini et al., 2011). However, propionate’s effects on health can be double-edged, with its pathophysiological roles varying depending on the health context. For instance, in propionic acidemia (PA)—a recessive metabolic disorder caused by mutations in the *PCCA* or *PCCB* genes—impaired propionyl-CoA metabolism leads to a host of metabolic alterations. The accumulation of propionate and the blockade of its metabolism have synergistically detrimental effects on various organs, with neurological dysfunctions being one of the most common complications in PA patients (Marchuk et al., 2023).

In this minireview, we summarize the current understanding of propionate sources, metabolism, and its pathological roles in neurological dysfunctions when its metabolism is impaired in PA.

## 2 Propionate source

In addition to dietary sources, SCFAs are primarily produced through the fermentation of dietary carbohydrates and certain amino acids in the intestine (Louis and Flint, 2017). The intestinal origin of propionate has been confirmed by many studies, including our recent data showing that propionate levels in the portal vein are approximately 50 times higher than that in circulating plasma in a mouse study (Wang et al., 2024). Further evidence comes from germ-free mice, which display extremely low propionate levels (70 times lower) in the portal vein compared to control mice, strongly indicating that the majority of propionate is derived from the microbiome (Wang et al., 2024).

**FIGURE 1:**
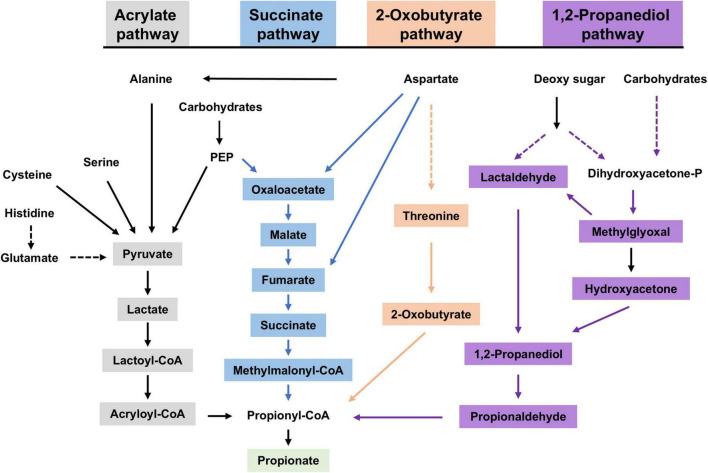
Propionate synthesis in microbiome. Four pathways (acrylate pathway in gray, succinate pathway in blue, 2-oxobutyrate pathway in orange, and 1,2-propanediol pathway in purple) lead to the synthesis of propionate in microbiome. PEP, phosphoenolpyruvate; Dihydroxyacetone-P, dihydroxyacetone phosphate.

The production of acetic, propionic, and butyric acid by the gut microbiome occurs in a molar ratio of approximately 3:1:1 (Hosseini et al., 2011). For a 70 kg human, the gut microbiome produces roughly 2 g of propionate per day (Morrison and Preston, 2016; Killingsworth et al., 2020), with concentrations reaching up to 10–30 mM in the proximal colon (Cummings et al., 1987). Propionate is produced through several metabolic pathways, including the succinate, acrylate, propanediol, and 2-ketobutyrate pathways (Figure 1; Hosseini et al., 2011; Louis and Flint, 2017). Among these, the succinate pathway is the most prevalent in the human gut microbiota (Kundra et al., 2024). Thus, propionate production by the microbiome can be influenced by diet, probiotics, and antibiotics. Interestingly, our recent findings show that a 23 h fasting significantly reduces microbiome-derived propionate (He et al., 2024).

Propionate can also be generated through the hydrolysis of propionyl-CoA. Acyl-CoA thioesterases are enzymes that catalyze the hydrolysis of CoA esters, converting them into free acids and CoA (Reaction formula 1) (Hunt and Alexson, 2002; Bai et al., 2024). These enzymes, also referred to as acyl-CoA hydrolases or palmitoyl-CoA hydrolases, help regulate intracellular levels of CoA esters together with carnitine acyl-CoA transferase, preventing the accumulation of acyl-CoAs, which cannot cross cellular membranes.


(1) A⁢c⁢y⁢l-C⁢o⁢A+H⁢O2→F⁢a⁢t⁢t⁢y⁢a⁢c⁢i⁢d+C⁢o⁢e⁢n⁢z⁢y⁢m⁢e⁢A

The hydrolysis of propionyl-CoA to propionate is not a reversible reaction of short-chain acyl-CoA synthetases (ACSSs). The activation of propionate to propionyl-CoA by a major isozyme (ACSS3) requires ATP (Yoshimura et al., 2017). These two opposing reactions complicate the assessment of propionate production from various metabolic sources. Consequently, using ^13^C-labeled propionate to measure the contributions of the microbiome, amino acids, and other sources to propionate production may be misleading due to the hydrolysis of propionyl-CoA to propionate (Thompson et al., 1990). Furthermore, overnight fasting may underestimate microbiome-derived propionate production, as indicated in our recent experiment with *Pcca*^–/–^(A138T) mice, an animal model for PA (He et al., 2024).

## 3 Physiological levels of propionate and propionyl-CoA

The intestinal microbiome produces significant amounts of SCFAs, including propionate (∼200 μM in the portal vein). However, circulating blood levels of propionate are much lower, approximately 0.4–5 μM, compared to acetate, which ranges from 50 to 200 μM (Bose et al., 2019; Kiasat et al., 2023). This difference in circulating levels of propionate and acetate is consistent with their corresponding acyl-CoA and acylcarnitine levels in tissues, where the C3/C2 (either acyl-CoA or acylcarnitine form) ratio is roughly 0.1 (Zhao et al., 2022).

The healthy liver efficiently metabolizes propionate, resulting in low systemic exposure in other organs (Wang et al., 2024). Given the liver’s high capacity to metabolize propionate, dietary propionate supplementation is not expected to substantially increase the circulating levels of propionate in healthy subjects. However, in metabolic conditions like PA, where liver metabolism of propionate is compromised, circulating propionate can rise markedly, reaching millimolar levels and leading to increased exposure in peripheral tissues (Wang et al., 2024).

## 4 Physiological functions of propionate

Propionate is considered beneficial to health and plays multiple physiological roles in the human body. For example, it promotes enteric smooth muscle contractions and modulates colonic motility (Mitsui et al., 2005). Additionally, propionate stimulates the synthesis of host defense peptides, which are critical in the body’s first line of defense against bacteria, fungi, parasites, and enveloped viruses (Sunkara et al., 2012). The metabolism of propionate is associated with glucose production and energy metabolism (Jones et al., 1997). Through a series of reactions, propionate is first converted to propionyl-CoA before ultimately being converted to succinyl-CoA (Deng et al., 2009; Jin et al., 2015; Wilson et al., 2017; Wang et al., 2018). Succinyl-CoA is a substrate in the TCA cycle and is further metabolized to oxaloacetate, which is a substrate for glucose synthesis. Thus, dietary propionate could impact the TCA cycle and gluconeogenesis (Brunengraber and Roe, 2006; Killingsworth et al., 2020).

Interestingly, propionate produced by the microbiome has been shown to improve various aspects of energy metabolism, such as reducing adiposity and body weight. This is achieved through the following mechanisms: (1) Propionate stimulates intestinal gluconeogenesis via a gut-brain neural circuit involving the fatty acid receptor FFAR3 (De Vadder et al., 2014). (2) Increased intestinal propionate has also been linked to reduced stress behaviors in mice (Burokas et al., 2017) and attenuated reward-based eating behaviors via striatal pathways in humans (Byrne et al., 2016). (3) Propionate exhibits antilipogenic and cholesterol-lowering effects by competitively inhibiting acetate uptake by liver cells (Delzenne and Williams, 2002) and reducing cholesterol biosynthesis (Arora et al., 2011). (4) Propionate may also help reduce obesity by promoting the secretion of PYY and GLP-1 hormones through binding and activating G-protein coupled receptors (GPR41 and GPR43) from enteroendocrine cells, which induce satiety, reduce energy intake, and promote weight loss (Arora et al., 2011; Killingsworth et al., 2020; Zhang et al., 2022).

Propionate has also demonstrated immune-modulatory and anti-inflammatory effects (Langfeld et al., 2021). Its regulation of the immune system occurs primarily through two key mechanisms: (1) Activation of G-protein coupled receptors for the SCFA receptor family, and (2) Inhibition of histone deacetylases (Tobin et al., 2021). Deficiency of propionate has been linked to an increased risk of asthma and allergies, underscoring its protective role in immune function (Bottcher et al., 2000; Ivashkin et al., 2019; Roduit et al., 2019).

## 5 Propionate metabolism in propionic acidemia

The first step of propionate metabolism is its activation into propionyl-CoA by ACSSs, specifically the ACSS3 isoform (Yoshimura et al., 2017). Propionyl-CoA, an anaplerotic substrate, enters the TCA cycle for complete metabolism. Propionate metabolism is highly efficient, particularly in the liver, maintaining circulating propionate at low levels (0.4–5 μM). However, this metabolism is disrupted when propionyl-CoA carboxylation is impaired, as seen in patients with PA. A reliable biomarker of PA is elevated propionate levels in both blood and urine, as observed in PA patients and mouse models, although propionate is often underreported due to the need for advanced analytical techniques (Wang et al., 2018). The precise mechanism by which PCC deficiency leads to elevated propionate remains unclear.

Our recent work demonstrated that ACSS3 activity in the liver is attenuated in *Pcca*^–^*^/^*^–^ (A138T) mice (Wang et al., 2018). As the liver is the primary organ in metabolizing propionate derived from the microbiome, the reduction in ACSS3 could contribute to the impaired metabolic disposal of propionate and its subsequent elevation. Another potential source of propionate is the hydrolysis of propionyl-CoA. Under normal conditions, intracellular propionyl-CoA levels are much lower than acetyl-CoA. Thus, the contribution of propionyl-CoA hydrolysis to propionate levels is likely minimal. However, in PA, when propionyl-CoA accumulates to levels comparable to acetyl-CoA, the contribution of propionyl-CoA hydrolysis to propionate production may increase. Despite this, it remains unclear how much propionate is produced from propionyl-CoA hydrolysis in PA. Addressing this question is critical to better understanding the pathophysiology of PA.

If propionyl-CoA hydrolysis were highly efficient, the accumulation of propionyl-CoA might not take place the primary issue, and the elevated propionate or other metabolites could instead be the main disease-causing factor. However, recent studies have shown that increasing coenzyme A synthesis via drug activators alleviates metabolic stress caused by propionyl-CoA accumulation (Subramanian et al., 2021; Subramanian et al., 2023). This suggests that even propionyl-CoA hydrolysis to propionate may occur, it is likely not a major contributor to overall propionate production or the regulation of propionyl-CoA levels.

## 6 Neurological dysfunctions associated with PA

While propionate has been shown to offer health benefits, its excess levels can be pathological. For instance, individuals with periodontal disease exhibit elevated levels of propionate in their saliva and may be at increased risk for developing Alzheimer’s disease (AD) (Killingsworth et al., 2020). Emerging evidence suggests that excess propionate, along with an increase in propionate-producing bacteria, may play a role in the development of dementia, particularly in AD (Arora et al., 2011).

In PA, where the PCC enzyme is impaired, propionate levels can rise significantly, reaching millimolar concentrations. The metabolic toxicity associated with these elevated propionate levels in PA has been well-documented (Shchelochkov et al., 1993; Marchuk et al., 2023), though the specific mechanisms behind the damage remain largely unclear. PA patients are subject to various complications (Marchuk et al., 2023). In addition to low muscle tone, cardiomyopathy, and pancreatitis, PA patients often present with a variety of neurological symptoms. These include psychomotor retardation, dystonia, developmental and speech delays, dementia, visual hallucinations, psychosis, seizures, stroke, coma, hypotonia, athetosis, optic neuropathy, acute hemiparesis, and other neurological impairments (Scholl-Burgi et al., 2009; Schreiber et al., 2012; Shuaib et al., 2012; Dejean de la Batie et al., 2014; Witters et al., 2016; AlGhamdi et al., 2018; Pfeifer et al., 2018; Almuqbil et al., 2019). Patients may also experience chronic psychological and cognitive sequelae, frequently leading to intellectual disability. Autism spectrum disorder has been reported in approximately 21% of PA patients (Witters et al., 2016; Cotrina et al., 2020).

Neurological impairments are observed more frequently in late-onset PA compared to early-onset cases (Jiang et al., 2022). Diagnosis Diagnostic imaging via magnetic resonance imaging typically reveals bilateral basal ganglia abnormalities, varying degrees of cisternal and sulcal widening, diffusion restriction, asymmetric atrophy, delayed myelination, corpus callosum dysplasia, volume loss in the vermis, gray matter vacuolization, and supratentorial white matter edema (Shuaib et al., 2012; AlGhamdi et al., 2018; Pfeifer et al., 2018; Jiang et al., 2022).

## 7 Pathological mechanisms of neurological dysfunctions in PA

### 7.1 Metabolic disruptions

**FIGURE 2:**
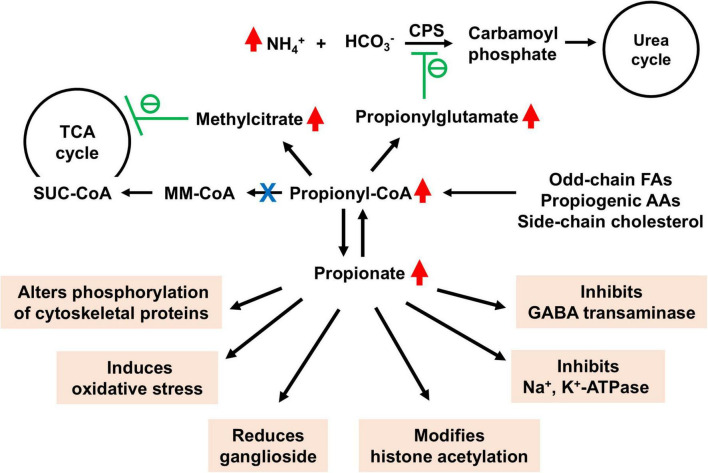
Detrimental effects of accumulated propionate and its metabolites in brain in propionic acidemia (PA). SUC-CoA, succinyl-CoA; MM-CoA, methylmalonyl-CoA; GABA, gamma-aminobutyric acid. Accumulated methylcitrate inhibits energy metabolism within the tricarboxylic acid (TCA) cycle, while elevated levels of propionylglutamate impair ammonium disposal via the urea cycle. The various toxic effects of accumulated propionate are highlighted in orange.

Figure 2 illustrates the current understanding of how propionate and its metabolites contribute to neurological dysfunctions observed in PA patients. The neurological manifestations in PA most likely stem from the metabolic consequences of elevated propionate and its metabolites (Scholl-Burgi et al., 2009; Schreiber et al., 2012). PA-related metabolic stroke, often caused by metabolic acidosis or hyperammonemia, contributes to acute neurological events (Almuqbil et al., 2019). Methylcitrate, a metabolite elevated in PA, has been shown to decrease glutamate oxidation by inhibiting glutamate dehydrogenase, as well as induce mitochondrial permeability transition. The reduction in glutamate oxidation and mitochondrial ATP generation are key factors in the neurological dysfunctions seen in PA (Amaral et al., 2016). In addition to inhibiting glutamate dehydrogenase, propionate and its metabolites also inhibit other TCA cycle enzymes such as pyruvate dehydrogenase, oxoglutarate dehydrogenase, and succinyl-CoA ligase, further compromising energy metabolism (Schreiber et al., 2012). These disruptions in energy metabolism particularly affect areas like the basal ganglia, which are highly energy-dependent and thus more vulnerable to damage from elevated propionate levels (Ji et al., 2022).

### 7.2 Persistent neurological damage

Despite lifelong dietary management, some PA patients develop late-onset bilateral optic neuropathy, suggesting a persistent pathological role of propionate in neurological dysfunctions (Williams et al., 2009). The neurotoxic effects of propionate have been observed in both animal models and human patients (Wyse et al., 1998; Colin-Gonzalez et al., 2015). For example, propionate has been shown to inhibit Na^+^, K^+^-ATPase activity in rat brain cortex, which may contribute to neurological dysfunctions in PA (Wyse et al., 1998). At concentrations equal to or lower than those found in the blood and brain of PA patients, propionate markedly affects the phosphorylation of cytoskeletal proteins in the cerebral cortex. Alterations in these proteins may disrupt cellular structure, leading to neurodegeneration (de Mattos-Dutra et al., 2000; de Almeida et al., 2006). Elevated propionate levels inhibit GABA transaminase, leading to the accumulation of GABA in the brain. This results in reduced neuronal activity, manifesting as lethargy (Morland et al., 2018).

Propionate also reduces ganglioside content in the cerebral cortex, which could contribute to brain damage in PA. This is similar to the effects seen with methylmalonic acid treatment, reinforcing the notion that brain damage is likely due to propionate rather than propionyl-CoA, which accumulates at much lower levels in methylmalonic acidemia (Trindade et al., 2002). High concentrations of propionate in the brain, as seen in animal models, lead to abnormal behaviors such as delayed habituation and repetitive motor activity (Brusque et al., 1999; Cotrina et al., 2020). These findings suggest that early postnatal propionate exposure results in long-term behavioral deficits.

### 7.3 Oxidative stress

Oxidative stress is another driver in PA-related neurological damage. Propionate has been shown to stimulate lipid peroxidation in brain tissue, leading to free radical generation that contributes to neurological dysfunctions (Fontella et al., 2000; Rigo et al., 2006; Ribas et al., 2010). In the hippocampus, chronic propionate exposure leads to spatial performance impairments, which are mitigated by ascorbic acid, an antioxidant (Pettenuzzo et al., 2002). Additionally, intrastriatal injection of propionate induces seizures and increases protein carbonyl content in the striatum, effects that are prevented by MK-801, an NMDA receptor antagonist, suggesting a role of NMDA receptors in PA associated oxidative damage and convulsions (Rigo et al., 2006).

### 7.4 Epigenetic modifications

Furthermore, propionate may alter neuronal gene expression by increasing histone acetylation (Nguyen et al., 2007). *In vitro* studies show that while propionate can be metabolized by glial cells, neurons lack this ability, potentially due to a lack of propionyl-CoA synthetase or mitochondrial transporters for propionate. However, exposure to propionate increases histone acetylation in both neurons and astrocytes, suggesting that neurons may be particularly vulnerable to chronically elevated propionate levels in PA (Nguyen et al., 2007). The epigenetic effects of elevated propionate levels remain largely unexplored, presenting a promising area for future research, as both propionyl-CoA and propionate could influence the propionylation, acetylation of proteins and histones and gene transcription (Chapman et al., 2015; Lagerwaard et al., 2021; Park et al., 2023; Yang et al., 2023).

In summary, the metabolic toxicity of elevated propionate in PA involves multiple pathological mechanisms, including mitochondrial dysfunction, oxidative stress, and disruptions in gene expression.

## 8 Therapeutic perspective targeting propionate metabolism in PA

Since chronically elevated propionate is a key factor in the neurological dysfunction associated with PA, treatment strategies should focus on reducing propionate synthesis and enhancing its metabolic disposal.

To reduce propionate synthesis, two main approaches have been reported: (1) Antibiotics: These are used to inhibit gut microbiota, thereby reducing microbial production of propionate, and (2) Carnitine supplementation: Propionyl-CoA can be converted to propionylcarnitine by carnitine acetyltransferase or to propionate by propionyl-CoA hydrolase, both of which help regulate cellular propionyl-CoA levels. Supplementing with L-carnitine supports the conversion of propionyl-CoA to propionylcarnitine, reducing propionate production.

To increase propionate disposal, several strategies have emerged. Firstly, pharmacological activation of CoA synthesis: This strategy has been shown to increase propionyl-CoA flux into the TCA cycle, reducing metabolic stress in PA mice (Armstrong et al., 2021; Subramanian et al., 2021; Subramanian et al., 2023). Secondly, liver transplantation: As the primary site of propionate metabolism, a healthy liver can metabolize 99% of propionate from the portal vein. Liver transplantation has been shown to improve neurological functions in PA patients by preventing metabolic decompensation and reducing propionate-related metabolites, such as methylcitrate and 3-hydroxypropionate (Nagao et al., 2013). Although no direct propionate data was reported, hepatic disposal of propionate is expected to improve. However, propionyl-CoA may still accumulate in other organs deficient in PCC, potentially leading to localized increases in propionate levels. Thirdly, mRNA enzyme replacement therapy: Recent advancements offer the potential to restore PCC activity and improve propionate metabolism. While this strategy shows promise as a future treatment option, current methods still face challenges with side effects (Jiang et al., 2020; Koeberl et al., 2024).

## 9 Summary

Propionate, a SCFA, is beneficial to health when present at moderate levels and metabolized normally. However, elevated propionate levels or impaired propionate metabolism in PA can lead to harmful effects and various complications, including neurological dysfunctions. Significant progress has been made in understanding the pathological mechanisms linking elevated propionate to neurological dysfunctions in patients with PA. However, several areas require further investigation, including propionate production from propionyl-CoA hydrolysis in the brain, cellular levels of propionate in the brain, and the impact of propionate/propionyl-CoA on protein and histone acylation.
